# Reply to Urbanization Theory for Growing Trend of Multiple Sclerosis Letter

**Published:** 2017-11

**Authors:** Rouhullah DEHGHANI, Mohammad Ali SAHRAIAN, Masoud YUNESIAN, Mostafa HADEII, Hamid Reza GILASI, Vahid KAZEMI-MOGHADDAM

**Affiliations:** 1. Social Determinants of Health (SDH) Research Center, Kashan University of Medical Sciences, Kashan, Iran; 2. Dept. of Environmental Health, School of Health, Kashan University of Medical Sciences, Kashan, Iran; 3. Neuroscience Institute, Tehran University of Medical Sciences, Tehran, Iran; 4. Center for Air Pollution Research, Institute for Environmental Research, Tehran University of Medical Sciences, Tehran, Iran; 5. Dept. of Environmental Health, School of Health, Sabzevar University of Medical Sciences, Sabzevar, Iran; 6. Dept. of Epidemiology, School of Health, Shahid Beheshti University of Medical Sciences, Tehran, Iran; 7. Dept. of Environmental Health, School of Health, Neyshabur University of Medical Sciences, Neyshabur, Iran

## Dear Editor-in-Chief

We read the letter by Zarghami and Hojjati, in which they considered some points regarding our article on the correlation between urbanization and multiple sclerosis (MS) disease (1). At the outset, the relationship observed in our study expressed totally as a possible relevance, as it always has been done in large-scale studies. The article also mentioned that urbanization had been used as an indicator of lifestyle changing, air pollution increase, etc. (2).

The significant relation between Urban Development or urbanization and increase of air pollution, modernization, lifestyle has been proven in many areas of the world (3, 4). These intertwined factors can cause many chronic diseases such as MS. On the other hand, the progress of society toward urbanization can increase social class and facilities related to health and medicine. This can be led to lower exposure of people to external risk factors, especially during childhood (5). Eventually, this process can increase the risk of chronic diseases’ prevalence such as MS among the elderly. In addition, urbanization is considered as one of the factors contributing to the increasing prevalence of MS (6).

Urban life has spread throughout the world the last two centuries and the number of cities been multiplied. These process, are also arising from penury, discrimination, and health risks from the environment (7). With increasing trend of urbanization in Iran and other countries during the past decades, the required standards of the modern city have not been met totally. According to the WHO, Iran, South Asia ranked worst in urban air pollution (8). In Iran, emissions of non-standard motors are higher compared to other Middle Eastern countries. Despite the middle-rank of Iran in case of industrialization, this country is defined as one of the high-polluted countries in terms of air pollution. The geographical position of most industrial cities in Iran is so that the air pollution levels are higher than standard limits in most days. Big and industrial cities in Iran are suffering from air pollution ascending from the speedy urbanization during the past 4 decades (9, 10). Furthermore, the contributions of demographic and biological factors are significant too. Therefore, unsustainable urbanization can be more influential than sustainable urbanization in MS increasing.

Thus, according to the maps from the National Register of MS Project, provinces with lower urbanization rates such as Sistan and Baluchestan and southern Khorasan have the lowest prevalence of MS. On the other hand, provinces such as Tehran and Isfahan with high urbanization rate have shown a high prevalence of multiple sclerosis (2).

Due to the large changes in demographic conditions and their significant relationship with environmental factors and lifestyle, demographic and genetic factors can be involved in the changes related to prevalence of MS in Iran.
